# CRISPR-Cas9 mediated knockout of the *white* gene in the bluetongue virus vector, *Culicoides sonorensis* (biting midge)

**DOI:** 10.1038/s41598-026-59276-2

**Published:** 2026-07-16

**Authors:** Katherine Nevard, Estela Gonzalez, Tim Harvey-Samuel, Christopher Sanders, Rafael A. Homem

**Affiliations:** 1 The Pirbright Institute, Ash Road, Pirbright, GU24 0NF Surrey UK; 2 Present Address: The Animal and Plant Health Agency, Woodham Lane, Addlestone, Surrey, KT15 3NB UK; 3 Present Address: Keele University, Keele, Newcastle, ST5 5BG UK

**Keywords:** *Culicoides sonorensis*, CRISPR-Cas9 gene editing, Intrathoracic injection, *White* gene knockout, DIPA-CRISPR, Biotechnology, Genetics, Microbiology, Molecular biology, Zoology

## Abstract

*Culicoides* biting midges are small blood feeding insects responsible for the transmission of important arthropod-borne viruses (arboviruses) such as bluetongue virus (BTV), Schmallenberg virus (SBV) and epizootic hemorrhagic disease virus (EHDV), which cause major losses to livestock production worldwide. *Culicoides sonorensis* is the primary vector of BTV in North America and one of the few *Culicoides* species to be colonised and reared in artificial conditions. Gene editing technology has been used to explore virus-vector interactions in other vector groups, particularly within mosquitoes. Despite the availability of a reference genome since 2018, to date there have been no reports of gene editing in *C. sonorensis*. Here, we report the first instance of gene editing in *C. sonorensis*, achieved by intrathoracic injection of adult females with Cas9 and sgRNAs targeting the *white* gene. We generated heritable mutations in the *white* gene which produced both white eye and red eye phenotypes and went on to establish a homozygous knockout line carrying a single mutation. We observed gene editing efficiencies of up to 12.3%, making this an efficient protocol for genetic manipulation of *Culicoides* biting midges, opening the door to functional genomics studies and the development of control strategies in these important and understudied disease vectors.

**Supplementary Information:**

The online version contains supplementary material available at 10.1038/s41598-026-59276-2.

## Introduction

*Culicoides* biting midges (Diptera: Ceratopogonidae) pose a significant risk to global livestock production as well as human health due to their ability to transmit several important arboviruses^1^. *Culicoides* biting midges are the vectors of bluetongue virus (BTV), the causative agent of bluetongue disease in wild and domestic ruminants such as sheep, cattle and deer in which it can cause ulcers, fever, lameness, abortion and death^2^. The global economic impact of BTV, driven by productivity losses and international trade restrictions on animals, has been estimated at about US$3 billion^3^. Outbreaks of BTV in northern Europe, including the UK, have resulted in losses amounting to tens of millions of Euros^4^. *Culicoides sonorensis* is the primary vector of BTV within its range in North America^5^. This species is also a vector of epizootic haemorrhagic disease virus (EHDV), that may cause high mortality in deer and cattle^6^. *Culicoides sonorensis* is one of only two *Culicoides* species for which colonies are maintained worldwide, and it is used as a model species for investigations of interactions between vector *Culicoides* and the viruses they transmit^7^.

Despite the need to understand and control *Culicoides*-borne diseases and the availability of the *C. sonorensis* genome sequence since 2018 [^8^], genetic tools for examining vector-virus interactions in *Culicoides* lag behind those available for other vectors such as mosquitoes^7^. Embryo microinjection of Cas9 and sgRNAs is commonly used to gene edit insects for functional genomic studies^9^. To date, however, there have been no successful gene editing attempts in *Culicoides*. Our previous attempts to gene edit *C. sonorensis* through embryo microinjection (unpublished) were unsuccessful due to very low embryo survival following injection, likely owing to small embryo size and sensitivity to mechanical trauma.

Gene editing by female adult intrathoracic injection was developed in 2018 as an alternative route to deliver Cas9 ribonucleoprotein (RNP) complexes into ovaries and developing oocytes, thereby bypassing the need to inject individual embryos^10^. The technique was initially developed in *Aedes* mosquitoes using recombinant Cas9 fused to a yolk -derived, vitellogenesis -related peptide ligand (P2C) to facilitate entry into developing oocytes^10^. Subsequent studies have shown that Cas9 entry into oocytes can occur without this ligand while still achieving practical gene editing efficiencies, using non-modified, commercially available Cas9, sgRNAs, and optional additions such as endosomal escape reagents (DIPA-CRISPR)^11^ or transfection agents (SYNCAS)^12^.

Gene editing by adult intrathoracic injection has now been demonstrated across diverse arthropods, including mosquitoes^10,13–15^, ticks^16^, moths^17^, and beetles^11^. It has proved particularly useful in taxa where embryo microinjection is challenging or unfeasible, e.g., whiteflies^18^, psyllids^19^, parasitoid wasps^20^, aphids^21^, stink bugs^22^, kissing bugs^23^, thrips^12^, mites^24^, predatory flower bugs^25^ and cockroaches^11^; see also review by Terradas et al.^26^.

Genetic knockout is a useful tool for functional genomics, overcoming the variable efficacy, transient action and lack of organism-wide effect of RNAi gene silencing techniques, and provides a starting point for developing genetic control technologies as established in other insect vectors, notably mosquitoes^27^. Genetic knockout using CRISPR/Cas9 has been achieved in a wide range of insect species, with genetic tools for *Culicoides* comparatively underdeveloped. This gap may reflect the practical difficulty of working with *Culicoides* species due to their small size and standard rearing protocols, which require a dedicated room per strain and large open larval trays with mechanical paddles to maintain water movement^28^.

To overcome these barriers and enable functional genetic research in *Culicoides*, we set out to establish a method for generating genetic knockouts in *C. sonorensis*. The *white* gene was selected as a target for knockout via intrathoracic injection as it is well characterised in *Drosophila melanogaster* and other insect species. In addition, mutations in *white* produce an easily identifiable white eye phenotype that has been used in numerous proof of concept gene editing studies across insect taxa including Diptera^29–33^, Hemiptera^18^, Lepidoptera^17^ and Coleoptera^34^.

Here, we report the first successful attempt to edit the genome of a *Culicoides* biting midge species, the BTV vector *C. sonorensis*. Facilitated by a small-scale rearing system that is low cost, easy to implement, and suitable for genetically edited *Culicoides*, we were able to establish a homozygous knockout strain with a null mutation in the *white* gene.

## Results

### Cas9 must be intrathoracically injected within 24 h of a blood meal

To determine whether intrathoracically injected Cas9 can be incorporated into developing oocytes of *C. sonorensis*, gravid females were injected with GFP-Cas9 at two time points: 24 and 48 h post blood meal, each with two concentrations of Cas9: 0.5ug/µl and 1ug/µl. Ovaries were dissected and examined under a fluorescence microscope 24 h after injection.

**Fig. 1:**
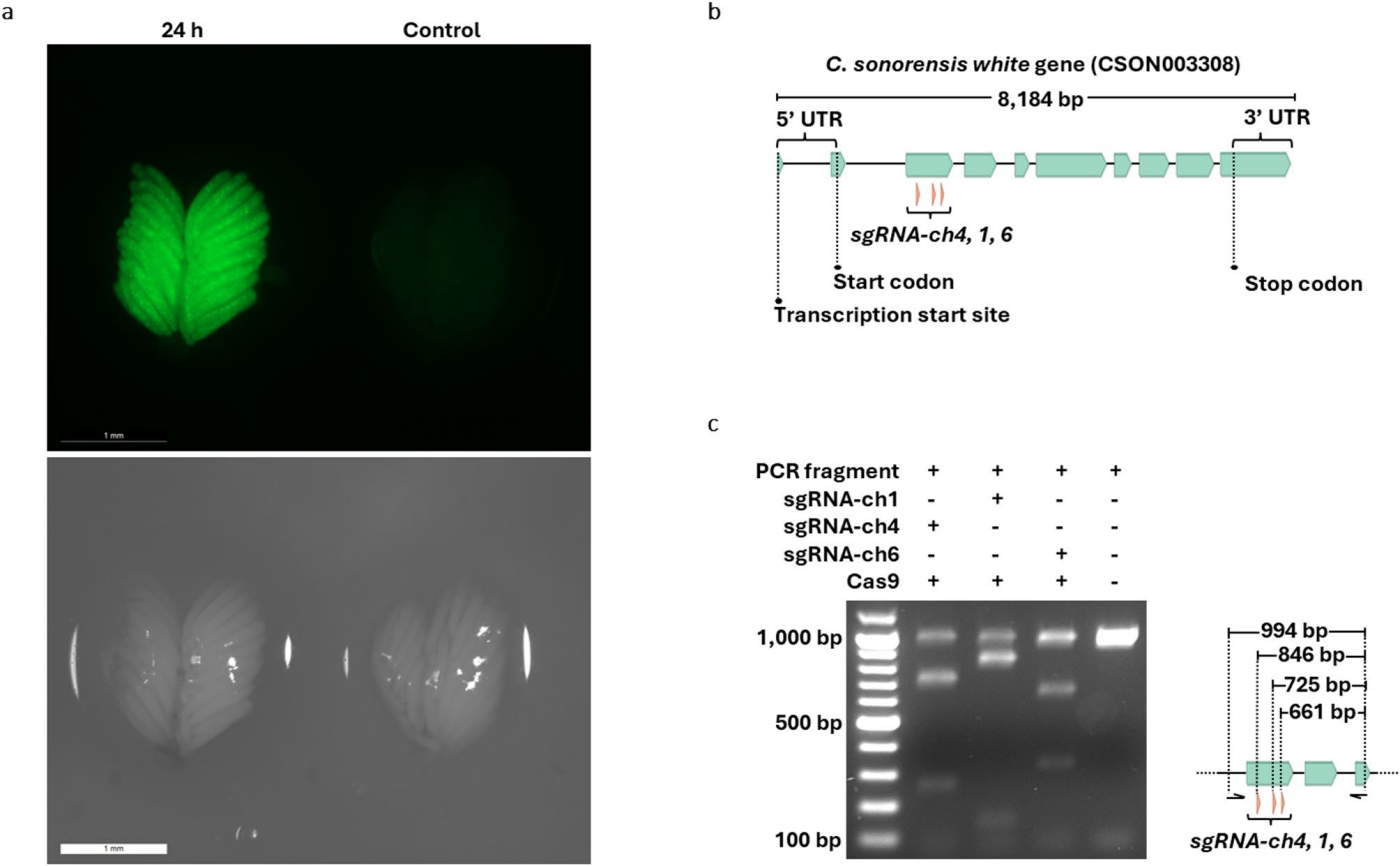
Intrathoracic Cas9 delivery to ovaries and molecular verification of sgRNA targeting within the annotated *white* gene of *C. sonorensis*. **(a)** Top - Fluorescence image of ovaries dissected from *C. sonorensis* female injected intrathoracically, 24 h after a blood meal, with GFP-Cas9 (left) and from control females injected with water (right). Bottom - equivalent brightfield image. **(b)** Schematic representation of the re-annotated *white* gene (introns not to scale) highlighting sgRNA binding sites within the third exon; **(c)** Left - In vitro digestion of a PCR fragment spanning gRNA target sites in exon 3 of the *white* gene using Cas9 in complex with sgRNA-ch1, 4 and 6. Undigested control shown on right. Primers Cs_white_001 and Cs_white_002 were used for PCR amplification (see Supplementary Table S3 for primer sequences). The image was cropped to remove blank spaces; the original uncropped image is provided in the supplementary material, Figure S3. Right - Schematic representation of the amplified region and expected fragment sizes after Cas9 digestions.

Strong GFP fluorescence was exhibited in dissected ovaries from females injected 24 h post blood meal, with paired ovaries showing equal levels of observed fluorescence (Fig. 1a). 97.1% and 96.3% of surviving females (*n* = 27; *n* = 34) showed fluorescence in their ovaries from 0.5 µg/µl and 1 µg/µl GFP-Cas9 injections respectively (Supplementary Table S1). In contrast, 0% and 5.0% of surviving females exhibited fluorescence in their ovaries when injected 48 h post blood meal with 0.5 µg/µl and 1 µg/µl GFP-Cas9, respectively (*n* = 20; *n* = 25) with only one female from the 1 µg/µl GFP-Cas9 injections displaying fluorescence. These findings demonstrate that intrathoracically delivered Cas9 can reach developing oocytes in *C. sonorensis*, but successful transfer requires injection within 24 h of a blood meal.

### Intrathoracic injections enable genome editing in *C. sonorensis*

We next investigated whether intrathoracic injections could deliver sgRNA-Cas9 ribonucleoprotein (RNP) complexes to edit the genome of *C. sonorensis*. As a proof of concept, we targeted the *white* gene, as mutations in this gene produce a visible eye phenotype in other insects.

First, we re-annotated the *white* gene (CSON003308) because the original annotation^8^ contained a misplaced stop codon in exon 6, resulting in a truncated protein of 434 amino acids compared to the typical ~ 700 amino acids observed in homologous proteins. To correct this, we aligned the mRNA sequence (CSON003308-1) with its orthologue in *C. brevitarsis* (LOC134834265), which encodes a 707-residue protein. The revised annotation revealed that the gene spans 8,184 nucleotides, comprises ten exons (including a non-coding first exon) (Fig. 1b), and encodes a 680-residue protein. Protein structural prediction models generated from the original and revised annotations confirmed that the re-annotated gene encodes for a full-length White protein whilst the original one lacked most of the transmembrane domains (Supplementary Figure S1a-b).

Using the updated annotation, we designed three sgRNAs targeting exon 3: sgRNA-ch1, sgRNA-ch4, and sgRNA-ch6, corresponding to the first, fourth, and sixth highest-ranked binding sites predicted by the CHOPCHOP web platform^35^(sgRNA sequences shown in Supplementary Table S2). All three sgRNAs were initially validated for activity in vitro. As shown in Fig. 1c, each sgRNA induced the expected cleavage of target PCR products when complexed with Cas9.

**Fig. 2:**
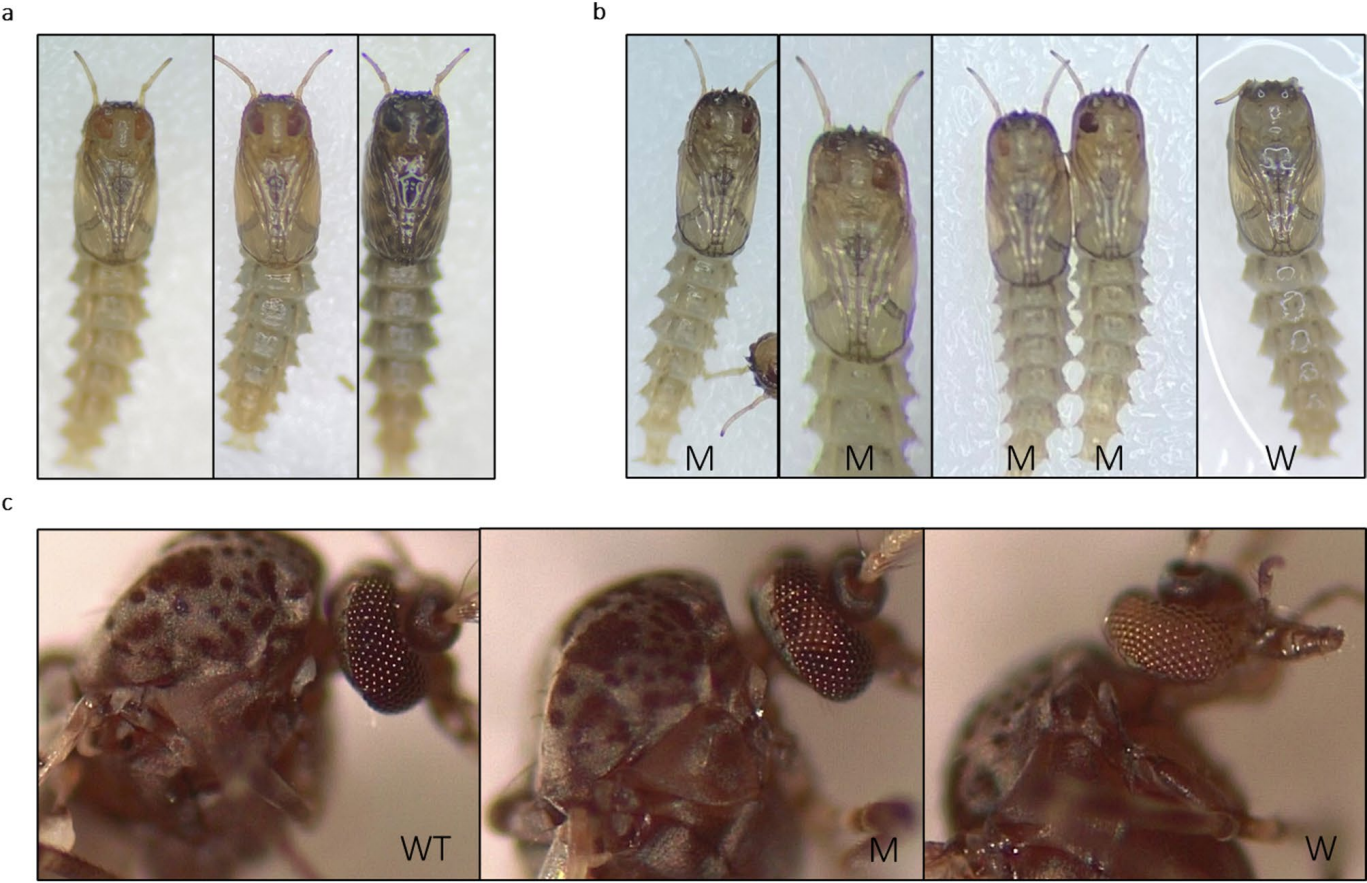
Eye pigmentation phenotypes in wild-type and gene-edited G₀ *C. sonorensis* pupae and adults. **(a)** Wild type *C. sonorensis* pupae with increasing eye pigmentation over time. Youngest pupa with light red eyes shown on the left, intermediate pupa with dark red eyes shown in the middle, and oldest pupa with black eyes shown on the right. **(b)** G₀ pupae with mosaic (M) and white (W) eye phenotypes. **(c)** G₀ adult midges with wild type (WT), mosaic (M) and white (W) eye phenotypes. G₀ pupae and adults are the progeny of females injected with Cas9-sgRNA complexes targeting the *white* gene.

Having confirmed sgRNA activity in vitro, we tested their efficiency in vivo by performing intrathoracic injections with varying concentrations of Cas9 and sgRNAs. We used a range of Cas9 concentrations (0.8ug/ul-3.3ug/ul), both similar to and in excess of GFP-Cas9 concentrations that were shown to transduce into the ovaries (0.5ug.ul-1ug/ul), in conjunction with various sgRNA concentrations, in order to capture a range of conditions which could improve gene editing efficiency. We assessed editing efficiency by scoring eye pigmentation in the progeny of injected females, termed G₀. The eyes of wild-type pupae gradually darken during development, transitioning from unpigmented white to fully pigmented black over approximately 48 h, with intermediate shades of yellow, orange, and red (Fig. 2a).

We identified G₀ pupae exhibiting distinct eye phenotypes, including mosaic and fully white eyes (Fig. 2b). These individuals were re-examined as adults to confirm phenotype persistence (Fig. 2c). Injection mixtures with the highest Cas9 concentration (3.3 µg/µl) produced the greatest number of mosaics and white eyed individuals (12.3%) but also resulted in the highest female mortality (75%) (Table 1).

**Table 1:** Comparison of gene editing efficiencies from injections with various Cas9-sgRNA injection mixes. Gene-editing efficiency is the percentage of gene edited pupae identified at G_0_ stage (mosaic and white eyes) out of the total number of G_0_ pupae screened.

Cas9 (µg/µl)	sgRNA (µg/µl)	Saponin (µg/µl)	Females injected	Female survivors	Mortality (%)	Females that laid eggs	Females that laid eggs (%)	Total eggs laid	Average eggs per female	Total G_0_s screened	G_0_s with mosaic eyes	G_0_s with white eyes	Gene-editing efficiency (%)
0.8	0.9	0.05	51	28	45	8	29	269	34	269	10	0	3.7
0.8	3.1	0.05	44	27	39	9	34	672	75	356	25	3	7.9
0.8	3.2	0.006	42	15	64	11	73	550	50	188	10	1	5.9
1.1	4.3	0.008	48	31	35	30	97	1838	61	616	20	1	3.4
1.7	6.4	0.011	32	18	44	17	94	841	50	350	12	3	4.3
3.3	12.8	0.023	24	6	75	5	83	280	56	130	16	0	12.3

### Cas9-induced mutations in the *white* gene are heritable

**Fig. 3:**
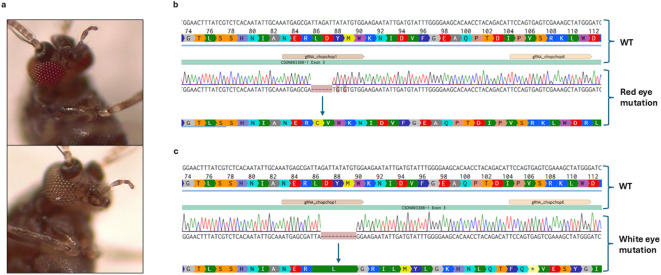
Phenotypic and molecular characterisation of CRISPR-induced mutations in the *white* gene of *C. sonorensis.* **(a)** G_1_ adults with red (top) and white (bottom) eyes. G_1_ progeny were generated by intercrossing G_0_ adults that presented mosaic and white eye phenotypes; **(b)** Sanger sequencing results highlighting nucleotide mutations and expected amino acid changes in the red eyed midges compared to wild type (WT). The deletion of six nucleotides and the substitutions of two other nucleotides, led to the deletion of two amino acids (L86 and D87) and the replacement of two others (Y88C and M89V); **(c)** Sanger sequencing results highlighting nucleotide and amino acid changes in the white eyed midges used to generate a strain with a single homozygous mutation. The deletion of ten nucleotides led to a frameshift in the amino acid sequence and the creation of a premature stop codon (*).

All G₀ adults displaying mosaic and white eye phenotypes were pooled in a single cage to allow mating and oviposition. Once G₁ eggs developed into pupae and adults, individuals were visually screened for eye colour. Three distinct eye phenotypes were observed among the G₁ progeny: black eyes, white eyes and red eyes (Fig. 3a). White eyed and red eyed G_1_ *Culicoides* were retained and separate cages of white eyed and red eyed individuals were set up to establish two populations.

To identify mutations associated with these phenotypes, genomic regions spanning the target locus were analysed by Sanger sequencing. A total of 10 G₁ *Culicoides*, three with red eyes and seven with white eyes, were examined. Because most mutants carried distinct allelic mutations on each homologous chromosome, sequencing chromatograms were analysed using the Inference of CRISPR Edits (ICE) tool^36^ to resolve and quantify edit profiles.

These analyses revealed that the red eye phenotype was associated with an in-frame mutation within the sgRNA-ch1 binding site. This mutation consisted of a six-nucleotide deletion and two adjacent substitutions, predicted to delete two amino acids (L86 and D87) and replace two others (Y88C and M89V) (Fig. 3b). Protein prediction models and superimposition of the wild-type and mutated proteins suggest that these amino acid changes may cause structural alterations to the cytoplasmic region of the protein, but do not seem to affect the transmembrane domains (Supplementary Fig. 1c-d). The red eye phenotype was observed when this mutation was homozygous or when in combination with a white eye associated, frameshift mutation.

The white eye phenotype was linked to multiple frameshift mutations, most located within or near the sgRNA-ch1 binding site. These included deletions of 4, 5, 10, 14, and 27 nucleotides. A single 21-nucleotide deletion was detected within the sgRNA-ch4 binding site, while no mutations were found within or near sgRNA-ch6. A complete list of mutations identified by Sanger sequencing and ICE analysis is provided in Supplementary Table S3.

To establish a homozygous white eyed strain with a single mutation, white eyed adult males (G₅) were individually placed in mating chambers with three blood-fed wild-type virgin females. After oviposition, males were removed and genotyped by Sanger sequencing. One male was identified with a homozygous 10-nucleotide deletion within the sgRNA-ch1 binding site. This mutation introduces a premature stop codon in exon 3, resulting in a truncated, non-functional White protein (Fig. 3c; Supplementary Fig. 1c). The heterozygous (*w*^+/- 10^) progeny of this male were intercrossed to generate homozygous individuals (*w*^*−* 10^), which were subsequently used to establish the final strain. This strain has since been maintained for over 15 generations.

## Discussion

Here, we demonstrated the feasibility of generating heritable mutations in the genome of *Culicoides sonorensis* using Cas9-sgRNA complexes delivered intrathoracically, and we showed that genotyping-guided genetic crosses can be applied to isolate specific alleles to produce a stable, homozygous, genome edited strain.

In terms of performance, our highest gene editing efficiency (12.3%) was achieved at the highest Cas9 and sgRNA concentration, although the lowest female survival (25%) was also observed at this concentration. This rate exceeds adult-injection gene editing efficiencies reported for most species (reviewed by^26^ ) but is significantly lower than values reported in *Blattella germanica* (up to 21.8%) [^11^], *Sogatella furcifera* (56.7%)^37^ and *Tribolium castaneum* (up to 71.4%)[^11^]. Our comparisons suggest scope for optimisation in *C. sonorensis*, notably through inclusion of branched amphipathic peptide capsules (BAPC) in the injection mix, which act synergistically with saponin to boost RNP delivery in other arthropods^12, 22^.

Furthermore, a recent study using the direct parental CRISPR (DIPA‑CRISPR) approach in *B. germanica* demonstrated, for the first time, successful knock‑in of a large DNA fragment via CRISPR‑mediated homology‑directed repair (HDR)^38^. The primary aim of that study was to tag an endogenous protein; however, this advance also opens the possibility of using this approach to generate transgenic tools that could improve genome‑editing efficiency and consistency. Examples include the generation of transgenic lines expressing Cas9 under the control of germline‑specific promoters. If such tools are transferable to *C. sonorensis*, they would be expected to substantially enhance editing efficiencies.

Our results also provide observations that may be relevant to the molecular interactions between subunits of ATP-binding cassette (ABC) transporters, a ubiquitous protein family involved in the translocation of diverse substrates across membranes^39^. In insects, pigment precursors are transported into eye pigment granules by heterodimeric ABC transporters composed of two distinct subunits: White-Brown (transporting red pigment precursors) and White-Scarlet (transporting brown pigment precursors). Consequently, loss of white function eliminates both pigment classes and results in a white-eye phenotype^40,41^.

Consistent with this established model, we identified five frameshift mutations predicted to truncate the White protein, each correlating with the white-eye phenotype. In addition, we identified an in-frame variant (L86-D87del; Y88C; M89V) associated with the red-eye phenotype. Structural prediction places these substitutions within the cytoplasmic region of the protein near the nucleotide-binding domain (NBD), which is required to bind and hydrolyse ATP and is important for transporter function and subunit interactions^42^. Based on this predicted localisation, we propose that these substitutions could affect transporter activity. One possible explanation is that the variant differentially impacts White-containing heterodimers, potentially reducing brown pigment precursor transport. However, this interpretation is based on structural inference and has not been directly tested. Prior work in *D. melanogaster* has demonstrated that mutations in different regions of White can have distinct phenotypic effects. For example, two mutations (H298N and G243S) in the NBD as well as one in an intracellular loop (G509D) reduced both red and brown pigments, whereas substitutions at the extracellular end of transmembrane helix 5 (G589E and F590G) preferentially reduced red pigment levels^43^. Previous studies also showed that changes in the C-terminal hydrophobic portion of White affected red pigment synthesis^44^.

These examples illustrate how specific domains contribute to transporter function and substrate specificity. To our knowledge, mutations in *white* have not previously been linked to selective impairment of White-Scarlet associated processes. Our observations raise the possibility that the variant identified in red-eyed *Culicoides* may differentially affect pigment pathways, potentially through altered subunit interactions. Further experimental work will be required to test this hypothesis directly.

Finally, the establishment of a *C. sonorensis* line with a visible marker phenotype will facilitate genome editing of loci that lack obvious phenotypes (e.g., immune‑related genes). Co-injecting sgRNAs targeting both the gene of interest and a marker locus (e.g., eye colour) into individuals heterozygous for the marker enables screening of progeny that display the marker phenotype, thereby enriching for edits at the target locus. This co‑CRISPR strategy has been validated in *D. melanogaster*^45^, achieving editing frequencies of up to ~ 70% in enriched groups. More recently this technique has been successfully applied to other species including *Aedes aegypt*^46^, and *Spodoptera frugiperda*^47^.

Altogether, the results presented here establish a versatile platform for CRISPR‑mediated gene knockouts in this important vector species. Combined with the rearing protocols outlined in the Methods, these advances provide a foundation for future functional genomics research, enhancing our ability to investigate, and ultimately manage traits that influence vector competence and pathogen transmission in this important vector group.

## Methods

### Culicoides rearing

*Culicoides* were reared in condition-controlled chambers at 27 C, 65% humidity and a 10/12 hour light/dark cycle with one hour of dawn and one hour of dusk. Adults were kept in Bugdorms (17.5cm^3^ and females were fed with defibrinated horse blood (TCS Biosciences) via a membrane feeding system (Hemotek) with the reservoir covered with Parafilm. One day post-blood feeding, cages were provided with an egg cup consisting of a 2oz plastic portion cup, containing wet cotton wool, covered with filter paper for the females to lay their eggs on (Supplementary Figure S2a). Eggs were collected 0–2 days after the provision of the egg cup, counted under dissection microscope and transferred to a rearing tray. Rearing trays were made with 800 ml tap water, left to stand overnight to dechlorinate, and inoculated with 200 ml of filtered water from an established rearing tray to act as a microbial seed. 0.5 ml of Oxoid Nutrient Broth Number 2 (Thermo Scientific), made up from 500 g dehydrated broth in 625 ml tap water, and 1.5cm^3^(1 Eppendorf) of grass meal and wheat germ mixture (2:1 ratio) were added to the tray on day one (day of setup) and day 10. To maintain water flow, air was pumped through a submerged tube, inserted through a hole in the lid of the tray (Supplementary Figure S2b). The rearing tray contained a piece of absorbent lint (Robinson Health Ltd) suspended over the tray so that the central region touched the water surface. Egg papers were transferred to the wet material, just above the water surface (Supplementary Figure S2c). This material also acted as a refuge for pupae, since C. *sonorensis* display a preference for pupation on or adjacent to a solid surface within the water. Pupae were collected daily, by submerging the material and allowing the pupae to float to the water surface, where they were collected with a plastic pipette onto a collection cup (same as egg cup). Pupae collection cups were put inside cages for the adults to eclose. Adults were provided with 10% sucrose solution *ad libitum*.

### GFP-Cas9 injections and ovary dissections

To assess the uptake of Cas9 into the ovaries and determine the optimum time window for injection, females, ~ 5–6 days post eclosion, were injected intrathoracically (Nanoject II, Drummond) under light CO_2_ anaesthesia, with 69-138nl of 0.5 µg/µl or 1 µg/µl GFP-Cas9 (IDT, Catalog # 10008100), 24 h and 48 h post blood feed. The total number of females injected is shown in Supplementary table S1. 24 h after injection, ovaries from surviving females were dissected out and viewed under a fluorescence microscope (Leica, M165 FC) with a GFP filter. Females were scored for the presence or absence of GFP fluorescence in the ovaries.

### Re-annotation of *C. sonorensis white* gene

The *white* gene (CSON003308) present in the reference genome of *C. sonorensis* (GCA_900258525.1) was identified as potentially misannotated based on the unusually small size of its predicted protein relative to homologous proteins in related species (Supplementary Figure S1a). This discrepancy appeared to be caused by the presence of a premature stop codon annotated in exon 6. To confirm this, the orthologous gene from another *Culicoides* species (*Culicoides brevitarsis*) was retrieved and used for comparison. The sequences and gene structures of *C. brevitarsis* (LOC134834265) and *C. sonorensis* (CSON003308-1) *white* transcripts were aligned using Benchling (https://benchling.com) and manually inspected for conserved exon boundaries and coding regions. Based on this analysis, it was concluded that the stop codon in exon 6 was likely erroneous. We then re-annotated the whole gene to reflect the correct exon boundaries and full-length coding sequence. This updated annotation (Fig. 1b) was subsequently used as template for downstream analysis.

### Protein structural modelling

Protein structure models were generated using the Benchling 3D structure prediction tool (Chai‑1 Model, version 0.61, Benchling, 2026) based on amino acid sequences corresponding to the misannotated *C. sonorensis white* gene, its corrected annotation, and *white* gene variants from red‑ and white eyed *Culicoides*. To compare structural differences, the predicted 3D structure of the protein from red eyed *Culicoides* was superimposed onto the wild‑type protein using the Pairwise Structure Alignment tool from the RCSB Protein Data Bank^48^.

### Design of sgRNAs targeting *white* and in vitro validation of Cas9 and sgRNA activity

sgRNAs targeting exon 3 of the re-annotated *white* gene (CSON003308) were designed using CHOPCHOP v3 and run through a BLAST search to check for off target genomic matches. Three sgRNAs were selected based on their on-target efficiencies and lack of off target genomic matches. These were commercially synthesised as single guide RNAs (crRNA and tracrRNA in the same molecule) (Sigma Aldrich). Target sgRNA cleavage sites are shown in Fig. 1C. Genomic DNA was extracted from wild-type (WT) adult *Culicoides* using the Phire Tissue Direct PCR Kit (Thermo Scientific) and PCR was carried out using the same kit to amplify the sgRNA targeting region with primers listed in Supplementary Table S4. Thermal cycling conditions consisted of an initial denaturation period of 98 °C for 1 min, followed by 35 cycles of 98 °C for 10s, 57 °C for 20s, and 72 °C for 40s, with a final extension at 72 °C for 5 min. An in vitro cleavage reaction was performed with each of the sgRNAs and SpCas9 (New England Biolabs) as per standard protocol (NEB #M0386) with the addition of RNase prior to the addition of proteinase K, following digestion, to remove sgRNAs and Cas9. Cleavage products were visualised by gel electrophoresis.

### Cas9 injections with *white* sgRNAs

Females (~ 5–6 days post eclosion), approximately 24 h post blood feed, were injected intrathoracically with 69-138nl of injection mix, using the Nanoject II Auto-Nanoliter Injector (Drummond), fitted with a quartz needle. Needles were pulled from quartz capillary tubes with filament (Length: 10 cm; OD: 1.0 mm; ID: 0.70 mm) (World Precision Instruments, catalogue number QF100-70-10) on a Sutter P2000 laser based micro-pipette needle puller (Sutter Instruments) using the following programme: HEAT = 720, FIL = 4, VEL = 40, DEL = 128, PUL = 134. Injection mixes were prepared by mixing glycerol free Cas9 (IDT) and equal concentrations of three sgRNAs targeting *white*, adjusted to various concentrations with nuclease free water (Table 1). This mixture was incubated at room temperature for 15 min to complex the Cas9 and sgRNAs and saponin solution was then added to the mix. Initial injection mixes with Cas9 concentrations of 3.3 µg/µl, 1.7 µg/µl, 1.1 µg/µl and 0.8 µg/µl were made by serial diluting the injection mix with the highest concentration. The resulting saponin concentrations in these injection mixes were 0.023 µg/µl, 0.011 µg/µl, 0.008 µg/µl and 0.006 µg/µl, respectively. Two subsequent injection mixes with Cas9 concentrations of 0.8 µg/µl were made with a saponin concentration of 0.05 µg/ml (see Table 1). Females were immobilised using CO_2_ delivered through a Fly pad connected to a Flowbuddy system (Flystuff, Genesee Scientific). Immediately after injection, females were transferred to a cardboard pot to recover. Approximately three hours later, females were immobilised again with CO_2_ and transferred to oviplates to allow for egg collection. Oviplates consisted of 24 well plates (Supplementary Figure S2d), with wells lined with filter paper and containing a detached 0.5 ml Eppendorf tube lid filled with 0.75% agar solution for egg deposition (Supplementary Figures S2e-f). Females were scored for survival approximately 24 h post injection. G_0_ eggs were counted and collected each day for up to two weeks and the number of egg-laying females were recorded (Table 1).

### Identification of mosaic and white eyed G_0_*C. sonorensis* and establishment of white eyed and red eyed populations

G_0_ eggs were transferred to trays by removing the agar on which eggs had been laid, from the Eppendorf lid and placing it onto the absorbent lint. G_0_ *Culicoides* were reared up to pupal stage and pupae were collected and screened each day under a dissection microscope for a mosaic or white eye phenotypes (Fig. 2b). Since eye pigmentation develops over the duration of the pupal stage (Fig. 2a), all pupae were re-screened the following day to confirm their mosaic or white eyed status. All mosaic and white eyed G_0_ adults were put into a cage where mating could occur. The cage was blood fed and G_1_ eggs were collected in an egg cup. G_1_ eggs were hatched and reared to pupal stage. Pupae were collected and screened daily for the presence of white eyes, which would indicate homozygous knockout of the *white* gene. Red eyed mutants were also identified at this stage and separate cages were set up with red eyed and white eyed individuals. White eye and red eye strains were maintained through several generations by collecting all pupae and adding them to a cage to mate and reproduce. Both strains displayed consistent eye phenotypes through the generations.

### Characterisation of *white* mutations

White and red eyed G_1_ adults were frozen individually at -80 °C and genomic DNA was extracted using the Qiagen DNeasy Blood and Tissue kit (Qiagen). A genomic region encompassing all three guide sites in exon three, was amplified by PCR using primers Cs_white_001 and Cs_white_002. (Supplementary Table S4). PCR products were purified using Monarch PCR and DNA cleanup kit (Monarch) and Sanger sequenced (Genewiz) with the above primers. Sequencing files were analysed manually and using the Inference of CRISPR Edits (ICE) tool^36^ to resolve individual mutations.

### Establishment of a *white* homozygous knockout line

To establish a homozygous colony with a single mutation, male G_5_ *Culicoides* were each crossed with three blood-fed female WT *Culicoides* in individual mating chambers. Following oviposition, males were removed and their genomic DNA was extracted (DNeasy Blood and Tissue kit, Qiagen) and amplified by PCR using primers Cs_white_001 and Cs_white_002. PCR products were purified using Monarch PCR and DNA cleanup kit (Monarch) and Sanger sequenced (Genewiz) to identify a single male that was homozygous for a 10 base deletion mutation at the gRNA-ch1 target site. This male was crossed with WT females, eggs collected from this male cross were hatched, and the resulting adults were inter-crossed. White eyed individuals were identified at pupal stage in the following generation and inter-crossed to generate a homozygous line.

## Supplementary Information

Below is the link to the electronic supplementary material.

Supplementary Material 1

## Data Availability

All data generated or analysed during this study are included in this published article (and its Supplementary Information files).
