# Supplementary material for: CRISPR-Cas9 mediated knockout of the white gene in the bluetongue virus vector, Culicoides sonorensis (biting midge)
Source: Sci Rep. 2026 Jul 16;16:19937. doi: 10.1038/s41598-026-59276-2 (PMC13376781; doi:10.1038/s41598-026-59276-2)
Supplement: Supplementary file 1 — Supplementary Material 1 [file 41598_2026_59276_MOESM1_ESM.pdf]

Supplementary Table S1. GFP visibility in dissected ovaries from females injected intrathoracically with GFP-Cas9, 24 or 48 hours after blood feeding. Ovaries were dissected out 24 hours post injection and viewed under fluorescence microscope with a GFP filter.

Injection (hours post BF)	GFP-Cas9 concentration (µg/µl)	Females injected	Survivors	Survival (%)	Survivors with GFP +ve ovaries	Survivors with GFP +ve ovaries (% of total survivors)
24	1	44	27	61.4	26	96.3
24	0.5	49	34	69.4	33	97.1
48	1	38	20	52.6	1	5.0
48	0.5	46	25	54.3	0	0.0

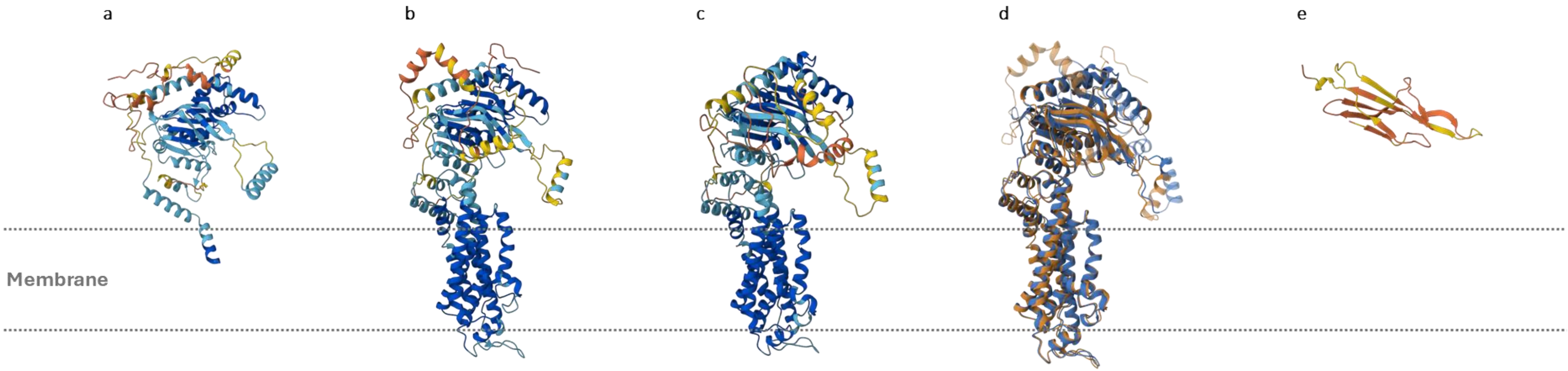

Supplementary Figure S1: Comparison of predicted structures for wild-type and CRISPR-edited variants of the *C. sonorensis* White protein. **a.** Protein structure prediction based on the originally annotated sequence of the *C. sonorensis white* gene. This model lacks most of the transmembrane regions of the protein. **b.** Predicted structure generated from the re-annotated sequence, restoring the predicted full-length protein. **c.** Predicted structure of a CRISPR-edited protein variant identified in midges with red eyes. This protein contains a two-amino acid deletion and two amino acid substitutions within the cytoplasmic region of the protein, leading to structural changes to this region. **d.** Superimposition of b and c, highlighting the structural changes caused by the mutations. **e.** Predicted structure of a CRISPR-edited protein variant identified in midges with white eyes. This protein contains a premature stop codon and lacks most of its predicted regions, leading to a small, low-confidence protein structure. All models were generated using the Benchling 3D structure prediction tool (Model Chai-1, version 0.61) and are shown in the same orientation for comparison. Structure superimposition was done using the Pairwise Structure Alignment tool RCSB PDB. Membrane annotations are displayed for reference.

Supplementary Table S2. gRNA sequences targeting of the *C. sonorensis white* gene.

gRNA name	Sequence
gRNA-ch1	ATGAGCGATTAGATTATATGTGG
gRNA-ch4	CTTACTAACGAAGAAGAACCAGG
gRNA-ch6	TCCAGTGAGTCGAAAGCTATGGG

Supplementary Table S3: Summary of mutations identified in G_1s at sgRNA target sites in the *white* gene. Negative values indicate deletion size in base pairs (bp) relative the reference sequence; + / + indicates no mutation detected at sgRNA target site; NR indicates that an allele could not be confidently resolved by Sanger sequencing and ICE analysis. sgRNAs listed in positional order.

Individual ID	Phenotype	sgRNA-ch4	sgRNA-ch1	sgRNA-ch6	Notes
1	White eyes	+ / +	-4 / -14	+ / +	Distinct heterozygous out-of-frame deletions
2	Red eyes	+ / +	-6 / -6	+ / +	Homozygous in-frame deletion plus two A-to-G substitutions within sgRNA-ch1 target site
3	Red eyes	+ / +	-6 / -10	+ / +	Distinct heterozygous in-frame and out-of-frame deletions plus two A-to-G substitutions within sgRNA-ch1 target site
4	White eyes	+ / +	-5 / -10	+ / +	Distinct heterozygous out-of-frame deletions
5	White eyes	+ / +	-4 / -4	+ / +	Homozygous out-of-frame deletion
6	White eyes	+ / +	NR / -10	+ / +	One allele not resolved, one out-of-frame deletion
7	White eyes	+ / +	-4 / -4	+ / +	Homozygous out-of-frame deletion
8	White eyes	-21 / -21	-27 / -27	+ / +	Homozygous in-frame deletion plus G-to-T and one T-to-C substitutions within sgRNA-ch4 target site and homozygous out-of-frame deletion within sgRNA-ch1 target site
9	White eyes	+ / +	-4 / -4	+ / +	Homozygous out-of-frame deletion
10	Red eyes	+ / +	-6 / -6	+ / +	Homozygous in-frame deletion plus two A-to-G substitutions within sgRNA-ch1 target site

Supplementary Table S4. Primers used in this study.

**Primer name**	**Sequence**
Cs_white_001	TATTGCAACATGATACGACTTC
Cs_white_002	ACATAACAAAAGGTTAGGATCC

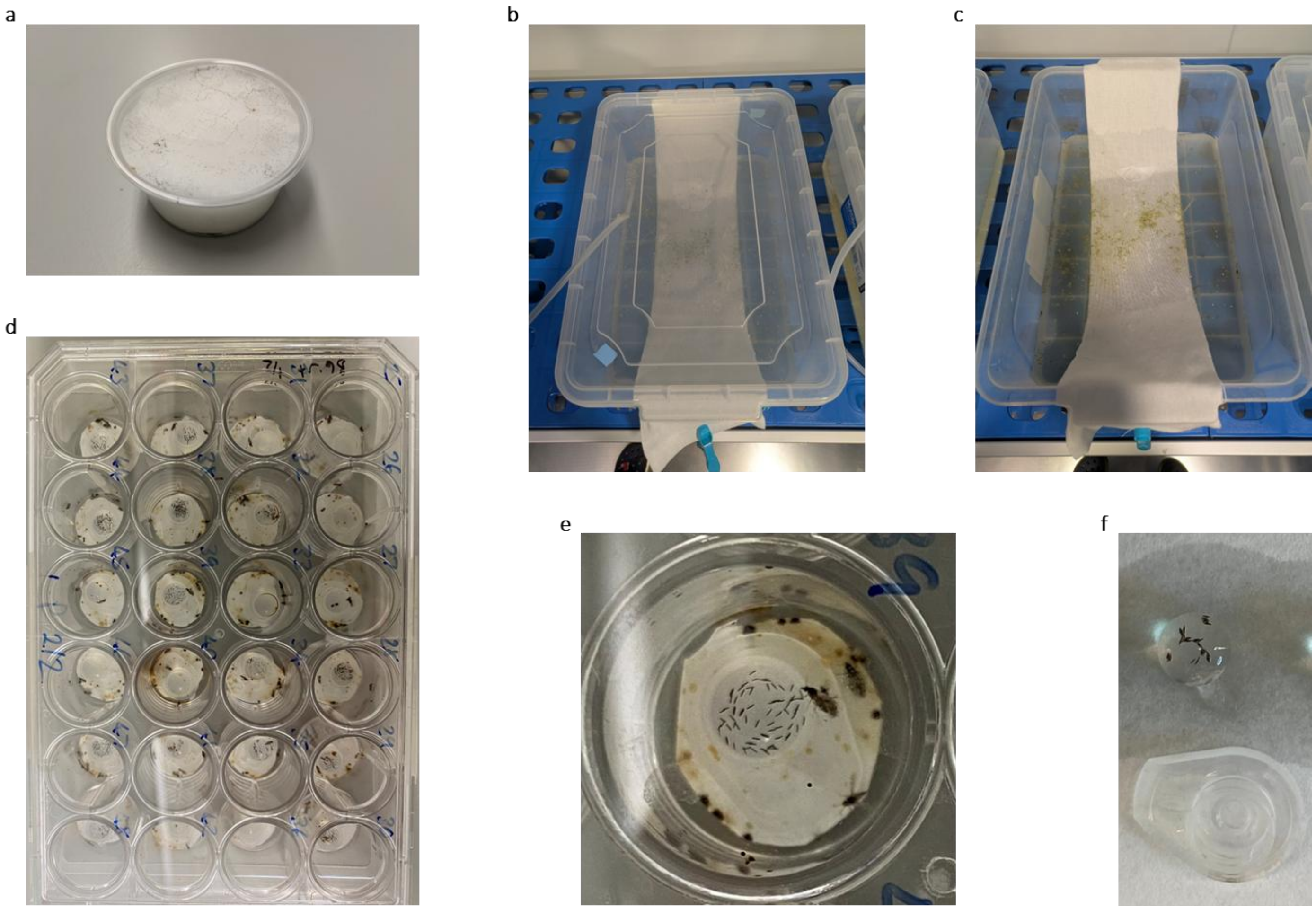

Supplementary Figure S2. a. Egg cup with deposited eggs used for rearing the gene-edited colony. **b**. Closed rearing tray with pumped air provided through submerged tube inserted through lid of tray. **c**. Rearing tray with section of egg paper placed on wet, suspended lint for hatching. **d**. Oviplate set up used to collect eggs from injected females. **e.** Well of an oviplate lined with filter paper and containing a detached 0.5ml Eppendorf tube lid filled with 0.75% agar solution for egg deposition. **f.** Agar substrate with deposited eggs removed from Eppendorf tube lid.

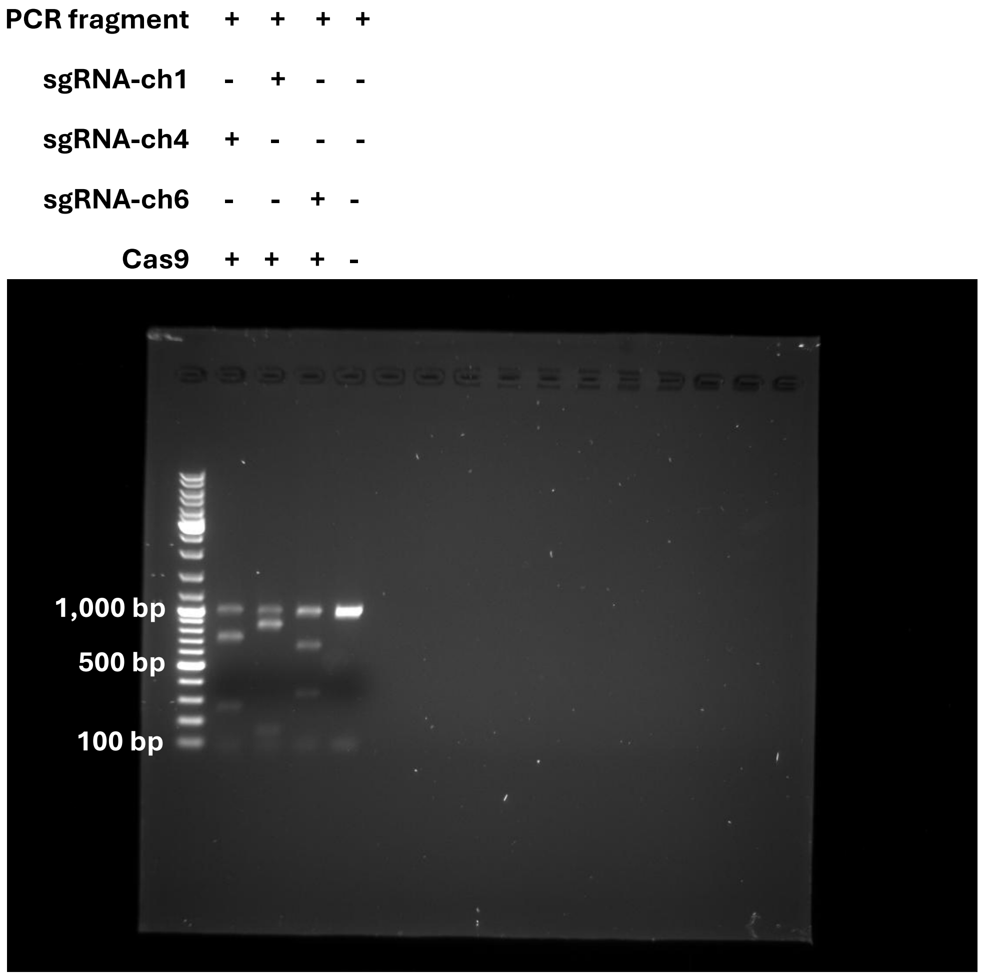

Supplementary Figure S3. Uncropped image of electrophoretic gel shown in Figure 1C - *In vitro* digestion of a PCR fragment spanning gRNA target sites in exon 3 of the *white* gene using Cas9 in complex with sgRNA-ch1, 4 and 6. Undigested control shown on right. Primers Cs_white_001 and Cs_white_002 were used for PCR amplification (see Supplementary Table S3 for primer sequences).
